# Analytical Compliance Equations of Generalized Elliptical-Arc-Beam Spherical Flexure Hinges for 3D Elliptical Vibration-Assisted Cutting Mechanisms

**DOI:** 10.3390/ma14205928

**Published:** 2021-10-09

**Authors:** Han Wang, Shilei Wu, Zhongxi Shao

**Affiliations:** 1 School of Mechanical Engineering, University of Shanghai for Science and Technology, 516 Jungong Road, Shanghai 200093, China; 2 School of Mechatronics Engineering, Harbin Institute of Technology, 92 West Dazhi Street, Harbin 150001, China; wushilei12@stu.hit.edu.cn (S.W.); shaozhongxi78@hit.edu.cn (Z.S.)

**Keywords:** compliance equations, high-precision positioning mechanism design, mechanical modeling, spherical flexure hinges

## Abstract

Elliptical vibration-assisted cutting technology has been widely applied in complicated functional micro-structured surface texturing. Elliptical-arc-beam spherical flexure hinges have promising applications in the design of 3D elliptical vibration-assisted cutting mechanisms due to their high motion accuracy and large motion ranges. Analytical compliance matrix formulation of flexure hinges is the basis for achieving high-precision positioning performance of these mechanisms, but few studies focus on this topic. In this paper, analytical compliance equations of spatial elliptic-arc-beam spherical flexure hinges are derived, offering a convenient tool for analysis at early stages of mechanism design. The mechanical model of a generalized flexure hinge is firstly established based on Castigliano’s Second Theorem. By introducing the eccentric angle as the integral variable, the compliance matrix of the elliptical-arc-beam spherical flexure hinge is formulated. Finite element analysis is carried out to verify the accuracy of the derived analytical compliance matrix. The compliance factors calculated by the analytical equations agree well with those solved in the finite element analysis for the maximum error; average relative error and relative standard deviation are 8.25%, 1.83% and 1.78%, respectively. This work lays the foundations for the design and modeling of 3D elliptical vibration-assisted cutting mechanisms based on elliptical-arc-beam spherical flexure hinges.

## 1. Introduction

Due to having good functional performances with proper design, surfaces with microstructural textures show their attractive application prospects in the fields of optics [1], biomedicine [2], tribology [3], mechanics [4], etc. Enormous amounts of attention have been paid to how to fabricate these surfaces with high efficiency and high accuracy by ultra-precision manufacturing. Compared with conventional mechanical forming methods, ultra-precision fast tool servo (FTS) [5,6,7] and elliptical vibration-assisted cutting (EVC) [8,9] are more effective ways to meet these requirements for surface texturing of difficult-to-machine materials. Since the flexible hinge can realize the transmissions of motion, force and energy through the elastic deformation of its flexible unit and has the advantages of high transmission accuracy, no friction, no lubrication, no gap, etc. [10,11,12], for FTS and EVC, high-precision positioning mechanisms based on the flexure hinge are generally used to fabricate micro-structured surfaces with high surface shape accuracy and working frequency [13,14]. Therefore, high-precision positioning mechanism design is of great significance for processing micro-structured industrial components with proper functional performance.

The mechanical modeling of flexure hinges is the basis of the kinetostatic modeling, even the analysis and design, of high-precision positioning mechanisms. Due to this great importance, the mechanical modeling of flexure hinges has been widely studied over the past two decades. The mechanical modeling of flexure hinges aims to model the compliance equations or compliance matrix that are used to describe the relationship between deformation and external loads. According to the existing literature, there are three main modeling methods for compliance equations/matrix: the one based on Mechanics of Materials, the one based on Castigliano’s Second Theorem, and the empirical one.

For the method based on Mechanics of Materials, Paros and Weisbord firstly established the mechanical model for right-circular type flexure hinges based on Mechanics of Materials [15]. Then, Smith developed this mechanical modeling method and derived compliance equations for elliptical type flexure hinges to reveal the relationship between deformation and external loads [16]. It is noted that the effect of shear forces on the compliance equations of flexure hinges is not considered during their mechanical modeling process. Chen et al. introduced the eccentric angle as an integral variable into the modeling process and derived generalized compliance equations for elliptical and quadratic-curve type flexure hinges. Then, this method was extended to the modeling of planar elliptical-arc-beam type flexure hinges [17,18]. Recently, Lu et al. formulated the compliance matrix of deep-notch elliptical flexure hinges based on the Mechanics of Materials without considering the effect of shear forces [19]. Unlike the previous studies, a numerical method is used to calculate the integrals during the modeling process.

On the other hand, Lobontiu introduced Castigliano’s Second Theorem into the mechanical modeling of flexure hinges and studied the compliance equations, motion accuracy and stress properties for all types of flexure hinges [20,21]. Considering the effect of shear forces, Shi et al. established the mechanical and accuracy models for all kinds of two-axis and three-axis flexure hinges based on Castigliano’s Second Theorem and studied the influence of structural parameters on the performance of flexure hinges [22]. Nguyen et al. used the same method to further analyze the compliance of a L-type straight-beam flexure hinge with the consideration of high-order shear effect and buckling effect [23].

In addition, a lot of practical empirical compliance equations are proposed by means of numerical methods. Smith et al. formulated the empirical equations of stiffness matrix in the functional directions of right-circular flexure hinges by finite element simulation [24]. Tian et al. presented the dimensionless empirical equations and graph expressions of filleted V-shaped, cycloidal and circular flexure hinges [25]. Li and Tuo used exponential models to formulate the empirical compliance equations for right-circular flexure hinges and the influence of stress concentration effect on the axial-compliance factor was further discussed [26,27].

In contrast to ultra-precision FTS and 2-D EVC, 3D EVC is more suitable for texturing surfaces with complicated microstructures [28]. According to the literature review, most of the compliance equations are derived for the design of two-dimensional planar flexure hinges, but few studies focus on the compliance equations for 3D, or spatial spherical flexure hinges. However, with the development of elliptical vibration-assisted actuators applied in 3D EVC, the demand for spatial spherical flexure hinges is increasing. It is necessary to develop the compliance equations for spatial spherical flexure hinges to briefly and accurately describe the relationship between deformation and external loads by generalized analytical compliance equations or compliance matrix. Meanwhile, from the reported literature, elliptical-arc type and straight-beam type are two basic types of flexure hinges. The elliptical-arc flexure hinge has high motion accuracy but relatively small rotation range, while the straight-beam flexure hinge has a large rotation range but poor motion accuracy. To achieve both high motion accuracy and large rotation range, the analytical compliance equations for a novel planar elliptical-arc-beam flexure hinge is derived in [18]. However, the analytical compliance equations for spatial ones has not been studied.

The contribution of this paper is to derive the generalized analytical compliance equations of spatial elliptical-arc-beam spherical flexure hinges so that designers can conveniently evaluate the kinetostatic performance of flexure hinges with all types of elliptical-arc-beam notch profiles, laying the foundation for high-precision positioning mechanism design of 3D EVC. Therefore, how to simply model the mechanical behavior of generalized spherical flexure hinges and deal with the formulation issue of the derived analytical equations are the two main tasks in this paper. To fulfill this, the mechanical model of a generalized flexure hinge is firstly established based on Castigliano’s Second Theorem. By introducing the eccentric angle as an integral variable, the compliance matrix of the elliptical-arc-beam spherical flexure hinge is formulated. Finite element analysis is carried out to verify the accuracy of the derived analytical compliance matrix. The novelty of this work is that the analytical compliance equations are theoretically extended to generalized elliptical-arc-beam spherical flexure hinges, which is promising to provide another design solution for spatial high-precision positioning mechanisms of 3D-EVC using spatial flexure hinges other than combining multiple planar flexure hinges. This is of great significance to the current state of industry.

## 2. Analytical Compliance Equations of Elliptical-Arc-Beam Spherical Flexure Hinges

### 2.1. Compliance Equations of Generalized Spatial Flexure Hinges

**Figure 1:**
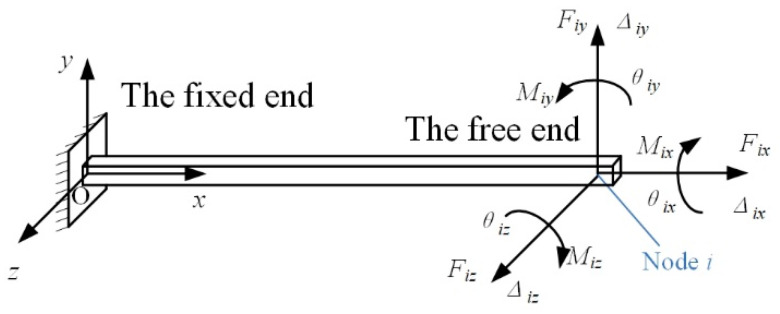
The coordinate system and load–displacement relationship of a spatial flexure hinge.

A spatial flexure hinge can be regarded as a section-variable cantilever beam with one side fixed. The coordinate system is defined at the fixed end as shown in Figure 1. When an external load vector F_i_ = [F_ix_, F_iy_, F_iz_, M_ix_, M_iy_, M_iz_]^T^ acts on node i at the free end of the flexure hinge, the flexure hinge will deform and a displacement vector ∆_i_ = [∆_ix_, ∆_iy_, ∆_iz_, θ_ix_, θ_iy_, θ_iz_]^T^ occurs.

The total elastic strain energy of the spatial flexure hinge comprises of the stain energy from tension/compression, shear, bending and torsion. The total stain energy of the flexure hinge U can be expressed as:

(1) $$U=\int_{0}^{L}\frac{F_{x}^{2}\left(x\right)}{2\mathrm{EA}(x)}\mathrm{dx}+\int_{0}^{L}\frac{\mu F_{y}^{2}\left(x\right)}{2\mathrm{GA}(x)}\mathrm{dx}+\int_{0}^{L}\frac{\mu F_{z}^{2}\left(x\right)}{2\mathrm{GA}(x)}\mathrm{dx}+\int_{0}^{L}\frac{M_{x}^{2}\left(x\right)}{2GI_{P}\left(x\right)}\mathrm{dx}+\int_{0}^{L}\frac{M_{y}^{2}\left(x\right)}{2EI_{y}\left(x\right)}\mathrm{dx}+\int_{0}^{L}\frac{M_{z}^{2}\left(x\right)}{2EI_{z}\left(x\right)}\mathrm{dx}$$

where L, A(x), E and G are the length of the flexure hinge, the area of the section at position x, the Young’s modulus and the shearing modulus of the material, respectively. Considering the flexure hinge may sometimes be abstracted as a short beam, the shearing coefficient μ is also introduced in Equation (1). For a short beam with circular section, the shear coefficient μ is 10/9. F_x_(x) is the axial force along x-axis. F_y_(x) and F_z_(x) are the shear forces along y-axis and z-axis. M_x_(x), M_y_(x) and M_z_(x) are the moments around the corresponding axes. For node i, the components of the external load vector are expressed as:

(2) $$\left\{ \begin{array}{l} F_{x}(x)=F_{\mathrm{ix}}\\ F_{y}(x)=F_{\mathrm{iy}}\\ F_{z}(x)=F_{\mathrm{iz}}\\ M_{x}(x)=M_{\mathrm{ix}}\\ M_{y}(x)=-F_{\mathrm{iz}}\left(L-x\right)+M_{\mathrm{iy}}\\ M_{z}(x)=F_{\mathrm{iy}}\left(L-x\right)+M_{\mathrm{iz}} \end{array}\right..$$

According to Castigliano’s Second Theorem, the partial derivative of the strain energy U to the external load F_i_ equals the displacement of the loading point **∆**_i_, which can be described as:

(3) $$\Delta_{i}=\frac{\partial U}{\partial F_{i}},$$

Combining Equations (1)–(3), the displacement of node i at the free end of the spatial flexure hinge can be expressed as:

(4) $$\Delta_{i}=C_{i}F_{i}=\left[\begin{array}{cccccc} C_{\Delta_{\mathrm{ix}}-F_{\mathrm{ix}}} & 0 & 0 & 0 & 0 & 0\\ 0 & C_{\Delta_{\mathrm{iy}}-F_{\mathrm{iy}}} & 0 & 0 & 0 & C_{\Delta_{\mathrm{iy}}-M_{\mathrm{iz}}}\\ 0 & 0 & C_{\Delta_{\mathrm{iz}}-F_{\mathrm{iz}}} & 0 & C_{\Delta_{\mathrm{iz}}-M_{\mathrm{iy}}} & 0\\ 0 & 0 & 0 & C_{\theta_{\mathrm{ix}}-M_{\mathrm{ix}}} & 0 & 0\\ 0 & 0 & C_{\theta_{\mathrm{iy}}-F_{\mathrm{iz}}} & 0 & C_{\theta_{\mathrm{iy}}-M_{\mathrm{iy}}} & 0\\ 0 & C_{\theta_{i}{}_{z}-F_{\mathrm{iy}}} & 0 & 0 & 0 & C_{\theta_{\mathrm{iz}}-M_{\mathrm{iz}}} \end{array}\right]F_{i}.$$

**C**_i_ is the compliance matrix at node i, the components of which are presented as follows:

(5) $$\left\{ \begin{array}{l} C_{\Delta_{\mathrm{ix}}-F_{\mathrm{ix}}}=\frac{4}{\pi E}\int_{0}^{L}\frac{1}{D^{2}\left(x\right)}\mathrm{dx}\\ C_{\Delta_{\mathrm{iy}}-F_{\mathrm{iy}}}=C_{\Delta_{\mathrm{iz}}-F_{\mathrm{iz}}}=\frac{4\mu }{\pi G}\int_{0}^{L}\frac{1}{D^{2}\left(x\right)}\mathrm{dx}+\frac{64}{\pi E}\int_{0}^{L}\frac{{\left(L-x\right)}^{2}}{D^{4}\left(x\right)}\mathrm{dx}\\ C_{\Delta_{\mathrm{iy}}-M_{\mathrm{iz}}}=C_{\theta_{\mathrm{iz}}-F_{\mathrm{iy}}}=-C_{\Delta_{\mathrm{iz}}-M_{\mathrm{iy}}}=-C_{\theta_{\mathrm{iy}}-F_{\mathrm{iz}}}=\frac{64}{\pi E}\int_{0}^{L}\frac{L-x}{D^{4}\left(x\right)}\mathrm{dx}\\ C_{\theta_{\mathrm{iz}}-M_{\mathrm{iz}}}=C_{\theta_{\mathrm{iy}}-M_{\mathrm{iy}}}=\frac{2G}{E}C_{\theta_{\mathrm{ix}}-M_{\mathrm{ix}}}=\frac{64}{\pi E}\int_{0}^{L}\frac{1}{D^{4}\left(x\right)}\mathrm{dx} \end{array}\right.$$

where C_m-n_ (m = ∆_ix_, ∆_iy_, ∆_iz_, θ_ix_, θ_iy_, θ_iz_, n = F_ix_, F_iy_, F_iz_, M_ix_, M_iy_, M_iz_) represents the compliance factor in the direction of m caused by the external load n and D(x) is the diameter variation of the corresponding circular section, which is a function of x.

### 2.2. The Notch Profile of Generalized Elliptical-Arc-Beam Spherical Flexure Hinges

**Figure 2:**
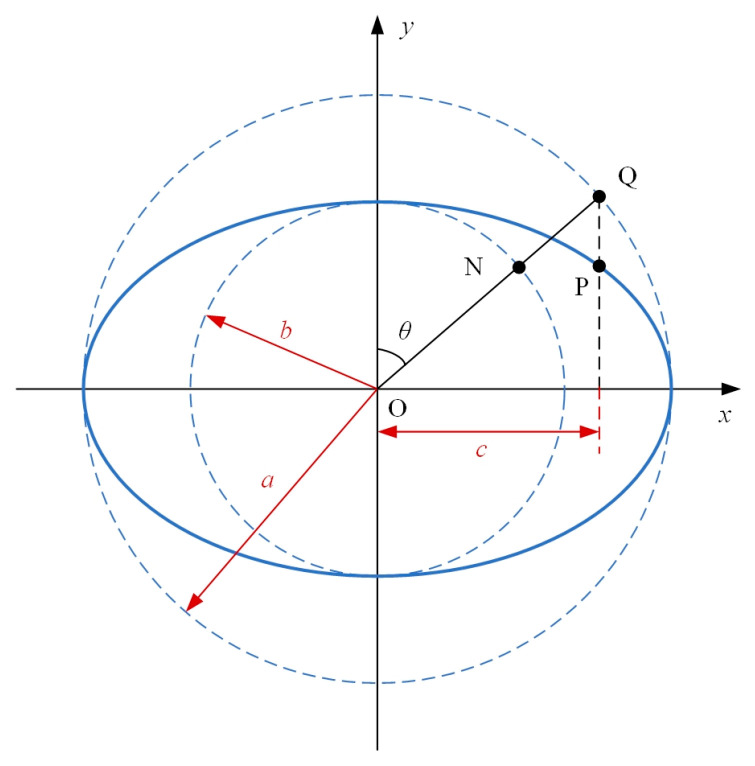
The eccentric angle of the ellipse.

To obtain the equations of the factors in the compliance matrix for elliptical-arc-beam spherical flexure hinges, the diameter variation of circular section D(x) in Equation (5) should be formulated. For an arbitrary point P on the ellipse of which the lengths of semi-major and semi-minor axes are a and b (Figure 2), its horizontal coordinate equals the projection of the corresponding point Q, which is on the circumscribed circle of the ellipse and determined by the eccentric angle θ, onto the x-axis, and its vertical coordinate equals the projection of the corresponding point N, which is on the inscribed circle of the ellipse with the same eccentric angle θ, onto the y-axis. The coordinates of P can thus be described as Equation (6)

(6) $$\begin{array}{cc} \left\{ \begin{array}{l} x_{P}=\mathrm{asin}\theta \\ y_{P}=\mathrm{bcos}\theta \end{array}\right.,\; & 0<\theta \leq \frac{\pi }{2} \end{array}$$

where a and b are also the radii of the circumscribed and inscribed circles of the ellipse, respectively.

According to the relationship between flexure hinges of different notch profiles in the relevant Reference [18], elliptical-arc spherical flexure hinges will degenerate to circular-arc ones when a is equal to b. They will also degenerate to circular ones when θ can reach up to π/2.

**Figure 3:**
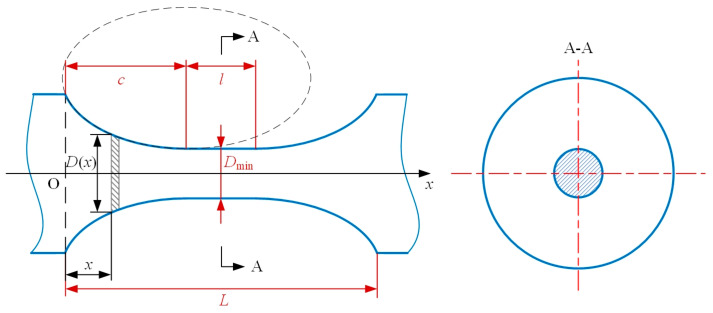
The diagram of the notch profile of spherical flexure hinges.

Figure 3 presents the notch profile of a generalized elliptical-arc-beam spherical flexure hinge. The notch profile can be determined by the notch length L, the lengths of semi-axes (a and b) of the ellipse and the diameter of the middle beam D_min_ which is the minimum diameter of the whole spherical flexure hinge. The notch length L includes the notch length of the elliptical-arc part c and the notch length of the middle beam part l, and L = l + 2c.

As shown in Figure 3, for arbitrary position x starting from the left-hand surface of the notch profile along the axis of the spherical flexure hinge, the diameter variation D(x) can be expressed as:

(7) $$D(x)=\{\begin{array}{cc} 2b\;+\;D_{\min}-\frac{2b}{a}\sqrt{a^{2}-{\left(c-x\right)}^{2}} & x\in \left[0,c\right]\\ D_{\min} & x\in \left[c,l+c\right]\\ 2b\;+\;D_{\min}-\frac{2b}{a}\sqrt{a^{2}-{\left(l+c-x\right)}^{2}} & x\in \left[l+c,L\right] \end{array}.$$

According to Figure 2 the relationship between the notch length of the elliptical-arc part and the semi-major axis of the ellipse is formulated as:

(8) $$c=\mathrm{asin}\theta_{m}$$

where θ_m_ is the maximum eccentric angle.

If D(x) in the form of Equation (7) is directly used, solving the compliance factors by Equation (5) needs to deal with complicated integrals, which makes the solutions cumbersome. In this paper, the variable substitution in Reference [29] is used to obtain the equations of the compliance factors

(9) x=c+asinθ.

By means of variable substitution, Equation (7) can be rewritten as:

(10) $$\{\begin{array}{cc} \begin{array}{l} D(\theta )=2b\;+\;D_{\min}-2\mathrm{bcos}\theta ,\\ D(x)=D_{\min},\\ D(\theta )=2b\;+\;D_{\min}-2\mathrm{bcos}\theta , \end{array} & \begin{array}{l} \theta \in [-\theta_{m},0]\\ x\in \left[c,l+c\right]\\ \theta \in [0,\theta_{m}] \end{array} \end{array}$$

and

(11) $$x=\{\begin{array}{cc} \begin{array}{l} c+\mathrm{asin}\theta ,\\ x,\\ l+c+\mathrm{asin}\theta , \end{array} & \begin{array}{l} x\in \left[0,c\right]\\ x\in \left[c,l+c\right]\\ x\in \left[l+c,L\right] \end{array} \end{array}.$$

The differentiation of Equation (11) yields:

(12) $$\mathrm{dx}=\{\begin{array}{cc} \begin{array}{l} \mathrm{acos}\theta d\theta ,\\ \mathrm{dx},\\ \mathrm{acos}\theta d\theta , \end{array} & \begin{array}{l} x\in \left[0,c\right]\\ x\in \left[c,l+c\right]\\ x\in \left[l+c,L\right] \end{array} \end{array}.$$

Supposing ζ = D_min_/2b, the diameter of the circular section of the elliptical-arc part at arbitrary θ can be expressed in polar coordinates as Equation (13):

(13) $$\{\begin{array}{cc} \begin{array}{l} D(\theta )=2b\left(1+\zeta -\cos\theta \right),\\ D(x)=2b\zeta ,\\ D(\theta )=2b\left(1+\zeta -\cos\theta \right), \end{array} & \begin{array}{l} \theta \in [-\theta_{m},0]\\ x\in \left[c,l+c\right]\\ \theta \in [0,\theta_{m}] \end{array} \end{array}.$$

### 2.3. Analytical Equations of the Factors in the Compliance Matrix

Combining Equations (5), (12) and (13), the factor equations of the compliance matrix to be solved are given as follows:

(14) $$C_{\Delta_{\mathrm{ix}}-F_{\mathrm{ix}}}=\frac{4a}{\pi E}\int_{-\theta_{m}}^{\theta_{m}}\frac{\cos\theta }{{\left[2b\left(\zeta +1-\cos\theta \right)\right]}^{2}}d\theta +\frac{4}{\pi E}\int_{c}^{l+c}\frac{1}{{\left(2b\zeta \right)}^{2}}\mathrm{dx},$$

(15) $$\begin{array}{ll} C_{\Delta_{\mathrm{iy}}-F_{\mathrm{iy}}} & =C_{\Delta_{\mathrm{iz}}-F_{\mathrm{iz}}}=\frac{4a\mu }{\pi G}\int_{-\theta_{m}}^{\theta_{m}}\frac{\cos\theta }{{\left[2b\left(\zeta +1-\cos\theta \right)\right]}^{2}}d\theta +\frac{4\mu }{\pi G}\int_{c}^{l+c}\frac{1}{{\left(2b\zeta \right)}^{2}}\mathrm{dx}\\ & +\frac{64}{\pi E}\int_{-\theta_{m}}^{0}\frac{{\left(l+c-\mathrm{asin}\theta \right)}^{2}\mathrm{acos}\theta }{{\left[2b\left(\zeta +1-\cos\theta \right)\right]}^{4}}d\theta +\frac{64}{\pi E}\int_{0}^{\theta_{m}}\frac{{\left(c-\mathrm{asin}\theta \right)}^{2}\mathrm{acos}\theta }{{\left[2b\left(\zeta +1-\cos\theta \right)\right]}^{4}}d\theta +\frac{64}{\pi E}\int_{c}^{l+c}\frac{{\left(l+2c-x\right)}^{2}}{{\left(2b\zeta \right)}^{4}}\mathrm{dx} \end{array}$$

(16) $$\begin{array}{ll} C_{\Delta_{\mathrm{iy}}-M_{\mathrm{iz}}} & =C_{\theta_{\mathrm{iz}}-F_{\mathrm{iy}}}=-C_{\Delta_{\mathrm{iz}}-M_{\mathrm{iy}}}=-C_{\theta_{\mathrm{iy}}-F_{\mathrm{iz}}}=\frac{64}{\pi E}\int_{-\theta_{m}}^{0}\frac{\left(l+c-\mathrm{asin}\theta \right)\mathrm{acos}\theta }{{\left[2b\left(\zeta +1-\cos\theta \right)\right]}^{4}}d\theta \\ & +\frac{64}{\pi E}\int_{0}^{\theta_{m}}\frac{\left(c-\mathrm{asin}\theta \right)\mathrm{acos}\theta }{{\left[2b\left(\zeta +1-\cos\theta \right)\right]}^{4}}d\theta +\frac{64}{\pi E}\int_{c}^{l+c}\frac{l+2c-x}{{\left(2b\zeta \right)}^{4}}\mathrm{dx} \end{array}$$

and

(17) $$C_{\theta_{\mathrm{iz}}-M_{\mathrm{iz}}}=C_{\theta_{\mathrm{iy}}-M_{\mathrm{iy}}}=\frac{2G}{E}C_{\theta_{\mathrm{ix}}-M_{\mathrm{ix}}}=\frac{64}{\pi E}\int_{-\theta_{m}}^{\theta_{m}}\frac{\mathrm{acos}\theta }{{\left[2b\left(\zeta +1-\cos\theta \right)\right]}^{4}}d\theta +\frac{64}{\pi E}\int_{c}^{l+c}\frac{1}{{\left(2b\zeta \right)}^{4}}\mathrm{dx}.$$

To further simplify the derivation, four intermediate variables N_j_ (j = 1, 2, …, 4) are defined by the integrals in Equations (14)–(17). Their expressions are shown in Appendix A. The analytical compliance factors can thus be expressed as follows:

(18) $$C_{\Delta_{\mathrm{ix}}-F_{\mathrm{ix}}}=\frac{4a}{\pi E}N_{1}+\frac{4}{\pi E}N_{2},$$

(19) $$\begin{array}{cc} C_{\Delta_{\mathrm{iy}}-F_{\mathrm{iy}}} & =C_{\Delta_{\mathrm{iz}}-F_{\mathrm{iz}}}=\left(\frac{4a\mu }{\pi G}+\frac{32a{\left(l+c\right)}^{2}+32ac^{2}}{\pi E}\right)N_{1}+\frac{128a^{2}l}{\pi E}N_{2}\\ & +\frac{64a^{3}}{\pi E}N_{3}+\frac{\mu }{b^{2}\zeta^{2}\pi G}+\frac{4}{3}\frac{{\left(c+l\right)}^{3}-c^{3}}{b^{4}\zeta^{4}\pi E} \end{array}$$

(20) $$C_{\Delta_{\mathrm{iy}}-M_{\mathrm{iz}}}=C_{\theta_{\mathrm{iz}}-F_{\mathrm{iy}}}=-C_{\Delta_{\mathrm{iz}}-M_{\mathrm{iy}}}=-C_{\theta_{\mathrm{iy}}-F_{\mathrm{iz}}}=\frac{32a\left(l+2c\right)}{\pi E}N_{1}+\frac{2l\left(l+2c\right)}{b^{4}\zeta^{4}\pi E},$$

and

(21) $$C_{\theta_{\mathrm{iz}}-M_{\mathrm{iz}}}=C_{\theta_{\mathrm{iy}}-M_{\mathrm{iy}}}=\frac{2G}{E}C_{\theta_{\mathrm{ix}}-M_{\mathrm{ix}}}=\frac{64a}{\pi E}N_{4}+\frac{4}{b^{4}\zeta^{4}\pi E}.$$

With these intermediate variables, the compliance matrix of generalized elliptical-arc-beam spherical flexure hinges can easily be obtained, avoiding the time-consuming integral operations during the analysis and design processes for the mechanism using spherical flexure hinges.

## 3. Results

### 3.1. Compliance Factors of Spherical Elliptical-Arc Flexure Hinges

Though few studies focus on the compliance matrix formulation of generalized spherical elliptical-arc-beam flexure hinges, analytical compliance equations of elliptical-arc spherical flexure hinges, which are the special cases, are reported. Therefore, the results of compliance factors of elliptical-arc spherical flexure hinges are presented first so that a comparison of the results calculated by other reported methods can be carried out to verify the correctness of the proposed analytical compliance equations. Elliptical-arc spherical flexure hinges of different minimum diameter D_min_ and notch types, including the elliptical, elliptical-arc, circular and right-circular types, are investigated. The geometric parameters of flexure hinges are listed in Table 1. The material of the spherical flexure hinges is structural steel, with Young’s modulus of 200GPa and Poisson’s ratio of 0.3. The results calculated by the proposed analytical compliance equations are shown in Table 2. The comparison will be discussed in Section 4.

### 3.2. Simulation Validation by FEA

To further verify the derived equations of the compliance factors for generalized elliptical-arc-beam flexure hinges, a simulation validation by FEA is conducted in this study. The compliance factors calculated by the derived equations are compared with those solved by the corresponding FEA.

For different notch types of spherical flexure hinges, the finite element models are established in ANSYS according to the tested geometric parameters. We investigated 18 hinge examples in this study, including the notch types of elliptical, right-circular, elliptical-arc, elliptical-beam, etc. The geometric parameters of the tested elliptical-arc-beam spherical flexure hinges are listed in Table 3. The flexure hinge is modeled by cutting axial-symmetric notch on the base cylinder. The length of the base cylinder is longer than that of the notch so that the rest part on each side can be used as an end block to apply loads and constraint conditions. In this simulation, the material of the spherical flexure hinges is 60Si2Mn, with Young’s modulus of 206GPa and Poisson’s ratio of 0.28.

**Figure 4:**
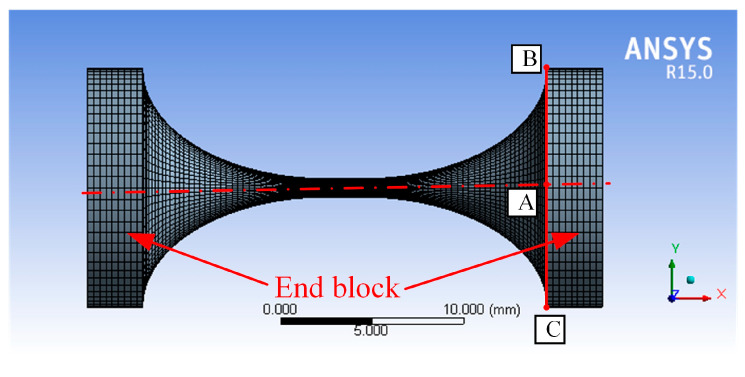
The finite element model of elliptical-arc spherical flexure hinges.

**Figure 5:**
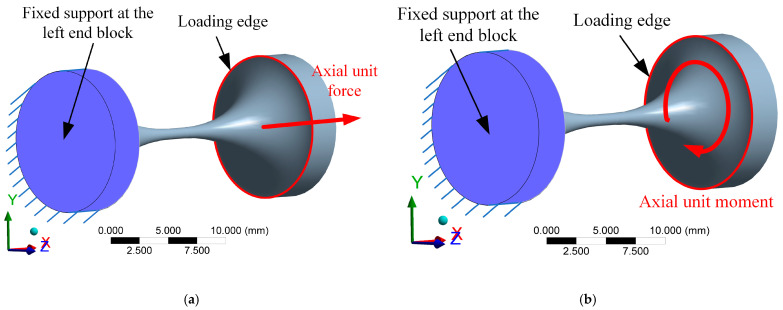
The constraint and load settings in the FEA: (**a**) Fixed support at the left end block and unit force applied onto the right end edge; (**b**) Fixed support at the left end block and unit moment applied onto the right end edge.

**Figure 6:**
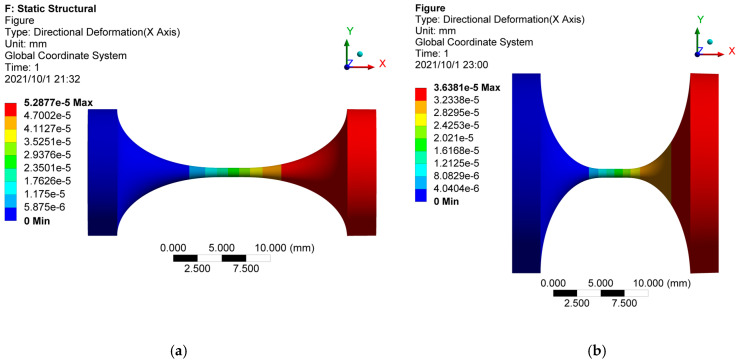

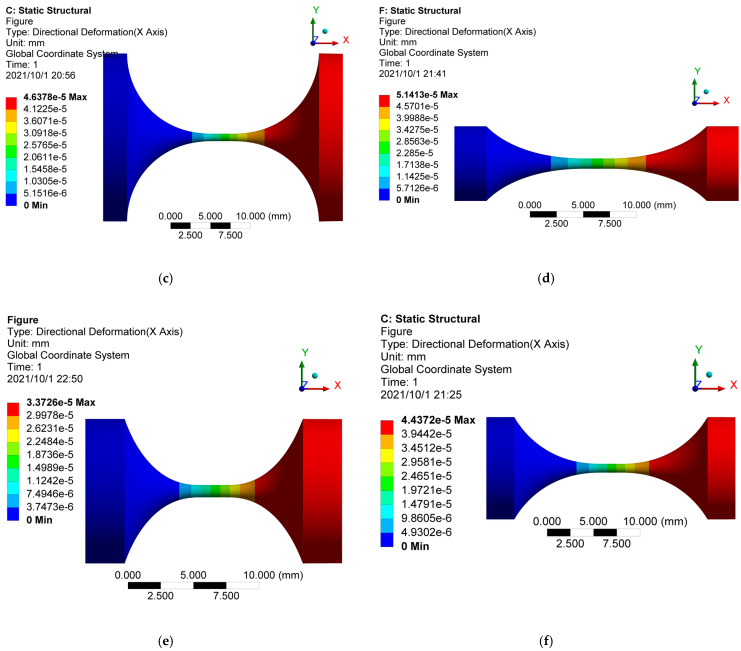
The x-directional displacement results of different notch types of flexure hinges: (**a**) Elliptical-beam; (**b**) Elliptical-beam; (**c**) Circular-beam; (**d**) Elliptical-arc-beam (**e**) Elliptical-arc-beam; (**f**) Circular-arc-beam.

The flexure hinge model is meshed by high-order three-dimensional 20-node elements SOLID186. To increase the mesh quality, the mesh refinement is carried out in the slender region of the hinge. The finite element model for hinge No.10 (elliptical-arc-beam type) is shown in Figure 4. Point A is the geometric center of the right end face of the flexure hinge. Point B and point C are the two ends of the section diameter at the right end face of the flexure hinge in the x–y plane. The boundary conditions, including constraint and load settings, are shown in Figure 5. In this FEA, the left end block in purple is set as the fixed support for all degrees of freedom. Loads of unit force (1N) in Figure 5a and unit moment (1N·mm) in Figure 5b are solely applied onto the right end edge (shown in red) of the flexure hinge based on the unit load method. Herein, taking the calculation process of C_Δix-Fix_ as an example, Figure 6a–f presents the x-directional displacement results of different notch types of flexure hinges (the length of the beam part is 2mm) under the application of the x-directional unit force (F_x_ = 1N). It is seen that to different extents, axial tensile deformation occurs to flexure hinges with different notch types, as the maximum x-directional displacement results range from 3.425 × 10^−5^ mm to 5.288 × 10^−5^ mm. The x-directional displacement of point A can be easily captured from these simulation results. Because the flexure hinge is applied by the unit load, C_Δix-Fix_ equals to the x-directional displacement of point A. Similarly, the displacements of A, B and C under different load conditions for all types of flexure hinges mentioned above are thus solved to calculate the positions and postures of the hinge’s right end according to the simple geometric relationship, respectively. Combined with the calculated positions and postures, the FEA results of the compliance factors by different loads can be obtained.

**Figure 7:**
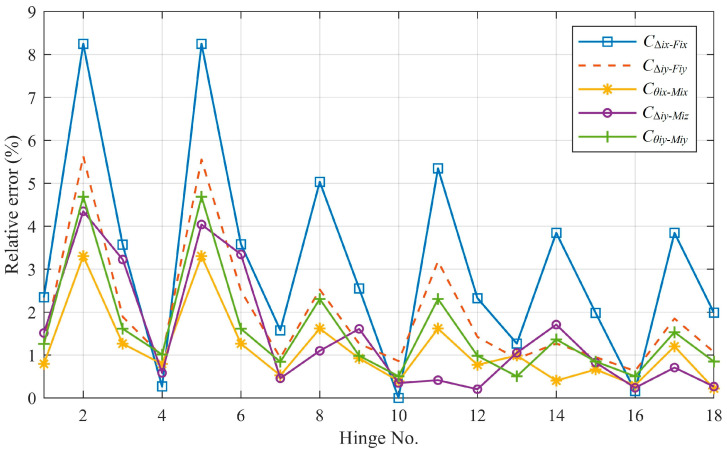
The relative error of compliance factors by the analytical method.

As listed in Table 4, for all notch types of flexure hinges, the compliance factors solved by the analytical method are compared with the FEA results, which can be regarded as the benchmark. The relative error is plotted in Figure 7. The maximum relative error is 8.25% and the average relative error is 1.83%. For flexure hinges of elliptical-arc notch and circular notch (a ≥ b, hinge No. 1, 3, 4, 6, 7, 9, 10, 12, 13, 15, 16 and 18), the relative error of each compliance factor is basically kept within 3.6%, while that of flexure hinges of elliptical-arc notch (a < b, hinge No. 2, 5, 8, 11, 14 and 17) is larger. It is also found that the longer the beam part of the notch is, the smaller the calculation error of the compliance factors is. The calculation error of C_Δix-Fix_ is bigger than that of the other compliance factors. The discussion of Figure 7 will be given in Section 4.

## 4. Discussion

As mentioned in Section 3, to verify the correctness of the derived analytical equations, a comparison with compliance equations derived by other methods should be carried out. It should be noted that the reason why the above geometric parameters (Table 1) and material properties are chosen for the calculation of compliance factors is that they are used in Reference [29], a typical work with a comparative verification of the existing equations, to verify the validity of analytical equations of elliptical-arc flexure hinges based on the beam theory. From Table 2, the results calculated by the proposed analytical compliance equations are almost the same as those listed in Reference [29] with a maximum relative error 1.3%. Although the analytical compliance equations are, respectively, developed based on the beam theory and Castigliano’s Second Theorem, there is no difference in essence in mechanics. That small error could be attributed to the calculation error during the integral process. According to Reference [29], it is indicated that the equations in this study are also much more accurate and stable than those developed by other existing equations reported in References [15,20]. Consequently, the analytical equations are correct for calculating the compliance factors for elliptic-arc spherical flexure hinges which, as mentioned above, are the special cases of generalized elliptic-arc-beam spherical flexure hinges.

From Figure 7, the compliance factors calculated by the analytical equations agree well with those solved by FEA since the maximum relative error is 8.25% and the average relative error is 1.83%, respectively. It should be noted that since the units and magnitudes of these compliance factors are different, the relative standard deviation is also used to verify the accuracy of the proposed equations. The relative standard deviation is 1.78%, indicating a good precision of the results. The reason why the relative error of compliance factors of elliptical-arc flexure hinges (a < b) are larger than those of flexure hinges of elliptical-arc notch (a ≥ b) is that the stress concentration of elliptical-arc notch (a < b) flexure hinge is more obvious than those of flexure hinges of other types, which affects the prediction accuracy of the corresponding compliance factors. Meanwhile, as the length of the beam part of the notch increases from 0 mm to 4 mm, the displacement of the flexure hinges is dominated by the deformation of the beam part, which can be perfectly predicted by the classical analytical beam model, while the influence of the stress concentration from the elliptical-arc part of which the section area varies decreases. This results in the trends of Figure 7 that the longer the beam part of notch is, the smaller the calculation error of the compliance factors is. Moreover, the calculation error of C_Δix_-_Fix_ is bigger than that of the other compliance factors, which is consistent with the analysis in Reference [30]. The reason is that in the theoretical mechanical model, the force between elements is assumed to be uniformly distributed, so that the deformation error between flexible elements caused by the concentrated force is not considered. Since there is a small relative error between the analytical solutions and the FEA solutions, the derived analytical compliance equations have enough accuracy to calculate the compliance factors and help designers to evaluate the kinetostatic behaviors of generalized elliptical-arc-beam spherical flexure hinges.

Consequently, according to the comparative results with the existing method and the FEA, the derived analytical equations are valid and correct for the computational analysis of generalized elliptical-arc-beam spherical flexure hinges, offering a convenient tool for analysis at early design stages of spatial compliance mechanisms for 3D-EVC.

## 5. Conclusions

This paper presents a formulation of analytical compliance equations for generalized elliptic-arc-beam spherical flexure hinges. Finite element analysis is carried out to verify the accuracy of the derived equations. The compliance factors calculated by the analytical equations agree well with those solved in the finite element analysis for the maximum and average relative error, which are 8.25% and 1.83%, respectively, and the relative standard deviation is 1.78%. Due to the small relative error and good precision between the analytical solutions and the FEA solutions, the derived analytical compliance equations have enough accuracy to calculate the compliance factors and help designers to evaluate the kinetostatic behaviors of generalized elliptical-arc-beam spherical flexure hinges. The analytical equations developed in this paper for the elliptic-arc-beam spherical flexure hinges are applicable for the computational analyses and designs of the spatial high-precision positioning mechanisms for 3D elliptical vibration-assisted cutting, providing researchers with another design solution for spatial high-precision positioning mechanisms of 3D-EVC using spatial flexure hinges other than combining multiple planar flexure hinges. Since the analytical compliance equations have been obtained, the modeling, analysis and optimization of a 3D elliptical vibration-assisted cutting mechanism based on the elliptical-arc-beam flexure hinges will be performed for high-precision surface texturing in our future work.

## Figures and Tables

**Table 1:** Geometric parameters of the elliptical-arc spherical flexure hinges.

Hinge No.	a (mm)	b (mm)	c (mm)	θm (°)	D_min_ (mm)	Notch Type
1	8.25	4	8.25	90	0.5	Elliptical
2	5	7	5	90	0.4	Elliptical
3	7	4	6.76	75	0.5	Elliptical-arc
4	4.5	6	3.90	60	0.4	Elliptical-arc
5	4	4	3.86	75	0.5	Circular
6	6	6	5.20	60	0.4	Circular
7	4	4	4	90	0.5	Right-circular
8	7	7	7	90	0.4	Right-circular
9	1	0.1	1	90	0.5	Elliptical
10	1	0.5	1	90	0.6	Elliptical
11	0.875	0.3	0.875	90	0.75	Elliptical

**Table 2:** The results calculated by the proposed analytical compliance equations.

Hinge No.	C_Δix-Fix_ (×10^−8^ m/N)	C_Δiy-Fiy_ (10^−5^ m/N)	C_θix__-Mix_ (rad/Nm)	C_Δiy-Miz_ (10^−3^ m/N)	C_θiy__-Miy_ (rad/Nm)
1	11.19	32.23	6.01	38.10	4.62
2	7.32	11.77	6.04	23.20	4.65
3	9.49	18.41	5.10	26.50	3.92
4	7.08	7.00	5.87	17.60	4.52
5	5.42	3.44	2.91	8.70	2.24
6	9.45	16.56	7.83	31.3	6.02
7	5.42	3.68	2.91	9.00	2.24
8	10.25	32.27	8.46	45.60	6.51
9	4.40	0.32	3.23	2.50	2.48
10	2.28	0.09	1.00	0.76	0.76
11	1.53	0.04	0.46	0.31	0.36

**Table 3:** Geometric parameters of the elliptical-arc-beam spherical flexure hinges in FEA.

Hinge No.	a (mm)	b (mm)	c (mm)	θm (°)	Dmin (mm)	l (mm)	Notch Type
1	10	6	10	90	1	0	Elliptical
2	6	10	6	90	1	0	Elliptical
3	10	10	10	90	1	0	Right-circular
4	10	6	8.66	60	1	0	Elliptical-arc
5	6	10	5.20	60	1	0	Elliptical-arc
6	10	10	8.66	60	1	0	Circular
7	10	6	10	90	1	2	Elliptical-beam
8	6	10	6	90	1	2	Elliptical-beam
9	10	10	10	90	1	2	Circular-beam
10	10	6	8.66	60	1	2	Elliptical-arc-beam
11	6	10	5.20	60	1	2	Elliptical-arc-beam
12	10	10	8.66	60	1	2	Circular-arc-beam
13	10	6	10	90	1	4	Elliptical-beam
14	6	10	6	90	1	4	Elliptical-beam
15	10	10	10	90	1	4	Circular-beam
16	10	6	8.66	60	1	4	Elliptical-arc-beam
17	6	10	5.20	60	1	4	Elliptical-arc-beam
18	10	10	8.66	60	1	4	Circular-arc-beam

**Table 4:** Comparison between the analytical solutions and the FEA solutions.

Hinge No.	C_Δix-Fix_ (×10^−8^ m/N)	C_Δiy-Fiy_ (10^−5^ m/N)	C_θix__-Mix_ (rad/Nm)	C_Δiy-Miz_ (10^−3^ m/N)	C_θiy__-Miy_ (rad/Nm)
1 (Analytical)	3.75	4.04	5.01	3.90	3.91
1 (FEA)	3.84	4.09	5.05	3.96	3.96
2 (Analytical)	1.78	0.67	2.34	1.10	1.83
2 (FEA)	1.94	0.71	2.42	1.15	1.92
3 (Analytical)	2.97	3.11	3.90	3.00	3.05
3 (FEA)	3.08	3.17	3.95	3.10	3.10
4 (Analytical)	3.73	3.06	5.01	3.40	3.91
4 (FEA)	3.74	3.09	5.05	3.42	3.95
5 (Analytical)	1.78	0.51	2.34	0.95	1.83
5 (FEA)	1.94	0.54	2.42	0.99	1.92
6 (Analytical)	2.96	2.34	3.90	2.60	3.05
6 (FEA)	3.07	2.40	3.95	2.69	3.10
7 (Analytical)	4.99	7.40	7.54	6.50	5.89
7 (FEA)	5.07	7.47	7.58	6.53	5.94
8 (Analytical)	3.02	1.93	4.87	2.70	3.81
8 (FEA)	3.18	1.98	4.95	2.73	3.90
9 (Analytical)	4.20	6.24	6.43	5.50	5.03
9 (FEA)	4.31	6.32	6.49	5.59	5.08
10 (Analytical)	4.97	5.77	7.54	5.70	5.89
10 (FEA)	4.97	5.82	7.57	5.72	5.92
11 (Analytical)	3.01	1.52	4.87	2.40	3.81
11 (FEA)	3.18	1.57	4.95	2.41	3.90
12 (Analytical)	4.20	4.85	6.43	4.90	5.03
12 (FEA)	4.30	4.92	6.48	4.91	5.08
13 (Analytical)	6.23	11.9	1.00	9.40	7.87
13 (FEA)	6.31	12.01	1.01	9.50	7.91
14 (Analytical)	4.25	3.89	7.40	4.60	5.78
14 (FEA)	4.42	3.94	7.43	4.68	5.86
15 (Analytical)	5.44	10.40	8.96	8.40	7.00
15 (FEA)	5.55	10.50	9.02	8.47	7.06
16 (Analytical)	6.20	9.48	1.007	8.40	7.87
16 (FEA)	6.21	9.54	1.01	8.42	7.91
17 (Analytical)	4.25	3.18	7.40	4.20	5.78
17 (FEA)	4.42	3.24	7.49	4.23	5.87
18 (Analytical)	5.43	8.32	9.00	7.50	7.00
18 (FEA)	5.54	8.41	9.02	7.52	7.06

## Data Availability

The datasets used or analyzed during the current study are available from the corresponding author on reasonable request.

## Nomenclature

All the parameters used in this paper are listed as follows:

**F**_i_	External load vector acts on node i at the free end of the flexure hinge
F_ik_	External force component of **F**_i_ with subscript k denoting its direction, k = x, y, z
M_ik_	External moment component of **F**_i_ with subscripts k denoting its direction, k = x, y, z
**∆**_i_	Displacement vector of node i at the free end of the flexure hinges resulted by **F**_i_
∆_ik_	Translation component of **∆**_i_ with subscript k denoting its direction
θ_ik_	Rotation component of **∆**_i_ with subscript k denoting its direction
x	Position coordinate along the flexure hinge
L	Length of the flexure hinge
A(x)	Section area of the flexure hinge, a function of x
E	Young’s modulus of material
G	Shearing modulus of material
μ	Shearing coefficient for a short beam with circular section
F_x_(x)	Axial force along x-axis of the flexure hinge, a function of x
F_k_(x)	Shear force along k-axis of the flexure hinge, a function of x, k = y, z
M_k_(x)	Moment around k-axis of the flexure hinge, a function of x, k = x, y, z
U	Strain energy of the flexure hinge acted by **F**_i_
**C**_i_	Compliance matrix of the flexure hinge at node i
C_m-n_	Compliance in the direction of m caused by the external load n, m = ∆_ix_, ∆_iy_, ∆_iz_, θ_ix_, θ_iy_, θ_iz_ and n = F_ix_, F_iy_, F_iz_, M_ix_, M_iy_, M_iz_
D(x)	Diameter variation of circular section, a function of x
a	Length of semi-major axis of ellipse
b	Length of semi-minor axis of ellipse
θ	Eccentric angle of ellipse
x_p_	Horizontal coordinate of point P on the ellipse
y_p_	Vertical coordinate of point P on the ellipse
D_min_	Diameter of the middle beam
l	Notch length of the middle beam part
c	Notch length of the elliptical -arc part
θ_m_	Maximum eccentric angle
ζ	Intermediate variable, ζ = D_min_/2b
N_j_	Intermediate variables for integral simplification, j = 1, 2, 3, 4

## Appendix A

The four integrals in the proposed equations are given as follows:

(A1) $$N_{1}=\int_{-\theta_{m}}^{\theta_{m}}\frac{\cos\theta }{D^{2}\left(\theta \right)}d\theta =\frac{1}{b^{2}\left(\zeta^{2}+2\zeta \right)}\left\{\frac{\arctan\left(\frac{\sqrt{\zeta +2}\tan\frac{\theta_{m}}{2}}{\sqrt{\zeta }}\right)}{\sqrt{\zeta^{2}+2\zeta }}+\frac{\left(\zeta +1\right)\tan\frac{\theta_{m}}{2}}{\left[\left(\zeta +2\right)\mathrm{ta}n^{2}\frac{\theta_{m}}{2}+\zeta \right]}\right\},$$

(A2) $$N_{2}=\int_{0}^{\theta_{m}}\frac{\sin\theta \cos\theta }{D^{4}\left(\theta \right)}d\theta =\frac{3\zeta +2}{96b^{4}\zeta^{3}{\left(\zeta +1\right)}^{2}}-\frac{\mathrm{co}s^{2}\theta_{m}\left(3\zeta -\cos\theta_{m}+3\right)}{96b^{4}{\left(\zeta +1\right)}^{2}{\left(\zeta -\cos\theta_{m}+1\right)}^{3}},$$

(A3) $$\begin{array}{ll} N_{3} & =\int_{-\theta_{m}}^{\theta_{m}}\frac{\mathrm{si}n^{2}\theta \cos\theta }{D^{4}\left(\theta \right)}d\theta \\ & =2\left(\frac{\mathrm{ta}n^{5}\frac{\theta_{m}}{2}}{8\zeta^{2}}+\frac{\left(\zeta +1\right)\mathrm{ta}n^{3}\frac{\theta_{m}}{2}}{8{\left(\zeta +2\right)}^{2}}+\frac{\tan\frac{\theta_{m}}{2}}{3\zeta \left(\zeta +2\right)}\right)/(\left(2b^{4}\zeta^{3}+12b^{4}\zeta^{2}+24b^{4}\zeta +16b^{4}\right)\mathrm{ta}n^{6}\frac{\theta_{m}}{2}\\ & +\left(12b^{4}\zeta^{2}+6b^{4}\zeta^{3}\right)\mathrm{ta}n^{2}\frac{\theta_{m}}{2}+\left(6b^{4}\zeta^{3}+24b^{4}\zeta^{2}+24b^{4}\zeta \right)\mathrm{ta}n^{4}\frac{\theta_{m}}{2}+2b^{4}\zeta^{3})\\ & +\arctan\frac{\left(b^{4}\zeta^{2}+4b^{4}\zeta +4b^{4}\right)\tan\frac{\theta_{m}}{2}}{b^{4}\left(\sqrt{\zeta \left(\zeta +2\right)}\right)\left(\zeta +2\right)}/8b^{4}{\left(\sqrt{\zeta \left(\zeta +2\right)}\right)}^{5} \end{array}$$

and

(A4) $$\begin{array}{ll} N_{4} & =\int_{-\theta_{m}}^{\theta_{m}}\frac{\cos\theta }{D^{4}\left(\theta \right)}d\theta ={\left(1+\cos\theta_{m}\right)}^{3}(\left(2\zeta^{3}+4\zeta^{2}+8\zeta +5\right)\frac{\mathrm{ta}n^{5}\frac{\theta_{m}}{2}}{2\zeta^{3}\left(\zeta +2\right)}+2(3\zeta^{3}\\ & +9\zeta^{2}+16\zeta +10)\frac{\mathrm{ta}n^{3}\frac{\theta_{m}}{2}}{3\zeta^{2}{\left(\zeta +2\right)}^{2}}+\left(2\zeta^{3}+8\zeta^{2}+16\zeta +11\right)\frac{\tan\frac{\theta_{m}}{2}}{2\zeta {\left(\zeta +2\right)}^{3}})/32b^{4}{\left(\zeta +1-\cos\theta_{m}\right)}^{3}\\ & +\frac{\left(4\zeta^{2}+8\zeta +5\right)\arctan\left(\frac{\sqrt{\zeta +2}\tan\frac{\theta_{m}}{2}}{\sqrt{\zeta }}\right)}{8b^{4}{\left(\sqrt{\zeta^{2}+2\zeta }\right)}^{7}} \end{array}$$
